# Polarizable Thiol–Ene Cross-Linked Nitrile Dielectrics for Stretchable Low-Voltage Neuromorphic Transistors with Acoustic Classification

**DOI:** 10.1021/acsami.5c18342

**Published:** 2026-01-05

**Authors:** Chang-Jing Liu, Shu-Wei Hsiao, Qun-Gao Chen, Qi-An Hong, Yen-Ting Lin, Chu-Chen Chueh, Chan-Tat Ng, Ting-Ting Chang, Seong H. Kim, Yu-Cheng Chiu, Wen-Ya Lee

**Affiliations:** † Department of Chemical Engineering and Biotechnology, 34877 National Taipei University of Technology, Taipei 10608, Taiwan; ‡ High-Value Biomaterials Research and Commercialization Center, National Taipei University of Technology, Taipei 10608, Taiwan; § Department of Chemical Engineering, National Taiwan University of Science and Technology, Taipei 10607, Taiwan; ∥ Department of Chemical Engineering and Materials Research Institute, 8082 The Pennsylvania State University, University Park, Pennsylvania 16802, United States; ⊥ Department of Chemical Engineering, 33561 National Taiwan University, Taipei 10617, Taiwan; # Department of Psychology and Research Center for Mind, Brain & Learning, 34913 National Chengchi University, Taipei 11605, Taiwan

**Keywords:** high-k dielectric materials, stretchable transistor, thiol–ene chemistry, nonvolatile memory, artificial synapse

## Abstract

A stretchable, high-*k* dielectric material based on thiol–ene-cross-linked nitrile-butadiene rubber (NBR) for synaptic transistors is demonstrated. We investigated NBR formulations cross-linked with three thiol cross-linkers. The thiol–ene-cross-linked NBR dielectrics achieve a high dielectric constant (*k* = 14.6), enabling low-voltage transistor operation (<5 V) and photopatterned capability. By comparing different thiol cross-linkers, we have found that more thiol groups facilitate higher charge mobility and larger hysteresis. The thiol–ene-cross-linked NBR dielectric-based transistor exhibited superior electrical properties, including a high mobility (0.42 cm^2^ V^–1^ s^–1^), a high ON/OFF ratio (10^4^), and a small threshold voltage (0.2 ± 0.4 V). More importantly, these devices effectively mimic synaptic functions. A large hysteresis, driven by dielectric polarization and enhanced by thiol introduction, was observed, particularly pronounced in NBR dielectric with multiple thiol-cross-linkers. The thiol–ene-cross-linked NBR device displayed superior short-term plasticity and long-term potentiation/depression, indicating its learning and memory capabilities. Encouragingly, the fully stretchable NBR transistor maintained good electrical performance, stable hysteresis, and essential synaptic behaviors even at 60% strain. As a practical demonstration for neuromorphic applications, the thiol–ene-cross-linked NBR device exhibited excellent acoustic classification performance, achieving recognition accuracy close to 99% even under mechanical deformation. In summary, the developed thiol–ene cross-linked NBR offers highly promising electronic properties for stretchable, low-voltage neuromorphic devices.

## Graphical abstract

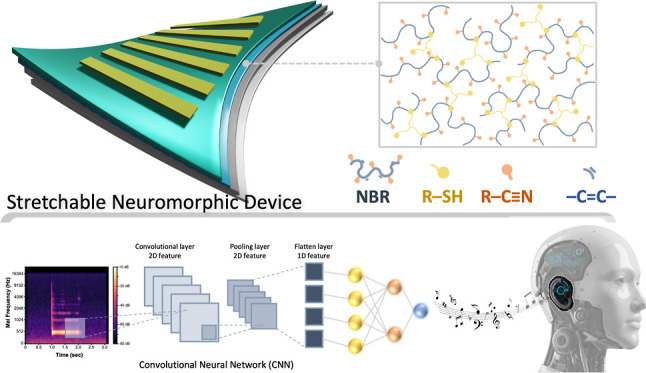

## 1 Introduction

The pursuit of artificial intelligence and the development of next-generation computing paradigms are driving intense research into neuromorphic devices that mimic synaptic functions of the brain. At the core of this research is the creation of artificial synapses, which simulate the behavior of biological synapses, to enable brain-inspired computing. Stretchable electronic materials have gained significant attention in this context due to their potential for creating stretchable and wearable neuromorphic systems.^1–8^ These materials must not only exhibit mechanical compliance but also possess the electrical properties required for synaptic functionality such as controllable conductivity, plasticity, and low-voltage operation.

Traditional silicon-based devices cannot meet the requirements of stretchable electronic devices, necessitating the exploration of new materials and device architectures. To date, various methods have been proposed to enhance the stretchability of semiconductor devices including rigid-island structures, buckling morphologies, and intrinsically stretchable materials. Compared with other methods, the development of intrinsically stretchable materials provides a greater potential as an ultimate solution for high-density stretchable integrated circuits. Although significant progress has been made in intrinsic stretchable semiconductors and conductors,^3–5,7–13^ developing stretchable dielectric materials with tailored properties remains a key challenge. In synaptic transistors, the dielectric layer plays a crucial role in modulating their electrical behavior and mimicking synaptic functions. Electronic properties, such as capacitance,^14,15^ driven voltage, polarizability, and the ability to control current hysteresis, are crucial for achieving synaptic plasticity and nonvolatile memory.

To identify suitable intrinsically stretchable dielectric materials, researchers investigated various elastomeric polymers, including polyurethane (PU),^16^ polydimethylsiloxane (PDMS),^12^ poly­(styrene-*block*-ethylene/butylene-*block*-styrene) (SEBS),^7,17^ and poly­(vinylidene fluoride-*co*-hexafluoropropylene) (PVDF–HFP).^9^ Ionic PVDF–HFP can provide ultrahigh electrochemical double-layer capacitance (250 nF cm^–2^), enabling low drive voltages (<5 V).^18^ However, due to slow ion diffusion, its high-frequency applications is limited, with capacitance significantly decreasing at frequencies exceeding 100 kHz. In contrast, ion-free high-performance polymer dielectric materials can operate at MHz frequencies, making them more suitable for high-speed devices.

In addition to high capacitance, the photocuring ability of stretchable polymer dielectric materials is important. Although polymer dielectric materials have demonstrated significant potential in the field of stretchable transistors, their sensitivity to common organic solvents poses a major challenge. Many polymer dielectric layers are easily soluble, rendering them incompatible with photolithography processes. This incompatibility hinders the mass production of stretchable electronic devices. To address this mass production issue, Bao’s group reported an intrinsically stretchable transistor array using a photo-cross-linked SEBS dielectric layer.^17^ To enhance solvent resistance, they modified the SEBS dielectric using azide-cross-linking chemistry. Under UV light activation, the azide groups react with C–H groups in the polymer chains. The same research group extended this approach to other elastomer polymers, including polyurethanes and conjugated polymers.^7^ To achieve monolithically integrated, low-voltage-driven stretchable transistor arrays, they utilized photoinduced azide cross-linking reactions to incorporate high-dielectric-constant elastomer dielectric material, nitrile-butadiene rubber (NBR), into the system.^8^ The permittivity of the NBR can significantly reduce the operation voltage. However, the strong polarizable characteristics of the NBR dielectric material led to significant current hysteresis in the devices. To mitigate this hysteresis effect, researchers prepared a trilayer structure composed of *n*-octadecyltrimethoxysilane (OTS)/SEBS/NBR for field-effect transistors. A photo-cross-linked SEBS layer modified with an OTS self-assembled monolayer was coated on the NBR layer to reduce threshold voltage shifts.

Recently, thiol–ene chemistry has emerged as a versatile tool for cross-linking and surface modification,^19^ enabling the preparation of a low-temperature processable, hydroxyl-free PVP dielectric layer.^20^ In this work, we utilized thiol–ene chemistry to cross-link NBR with three different thiol cross-linkers, including ethylene glycol bis-mercaptoacetate, trimethylolpropane tris­(3-mercaptopropionate), and pentaerythritol tetrakis­(3-mercaptopropionate), denoted as 2S, 3S, and 4S, respectively (Figure 1a). Different cross-linkers can manipulate the cross-linking density and polarizability of the dielectric layer. The resulting tunable polarization properties pave the way for developing photo-cross-linked high-dielectric-constant NBR-based stretchable transistors, which enables fabrication of artificial synapses and nonvolatile memory devices. The photo-cross-linking properties of these transistors make them suitable for large-scale array production. This work explores the development of a novel high-*k* polymer elastomer dielectric, demonstrating its capacity for mimicking neuronal synaptic behavior in stretchable electronic applications tailored for the artificial auditory sensory system.

**Figure 1:**
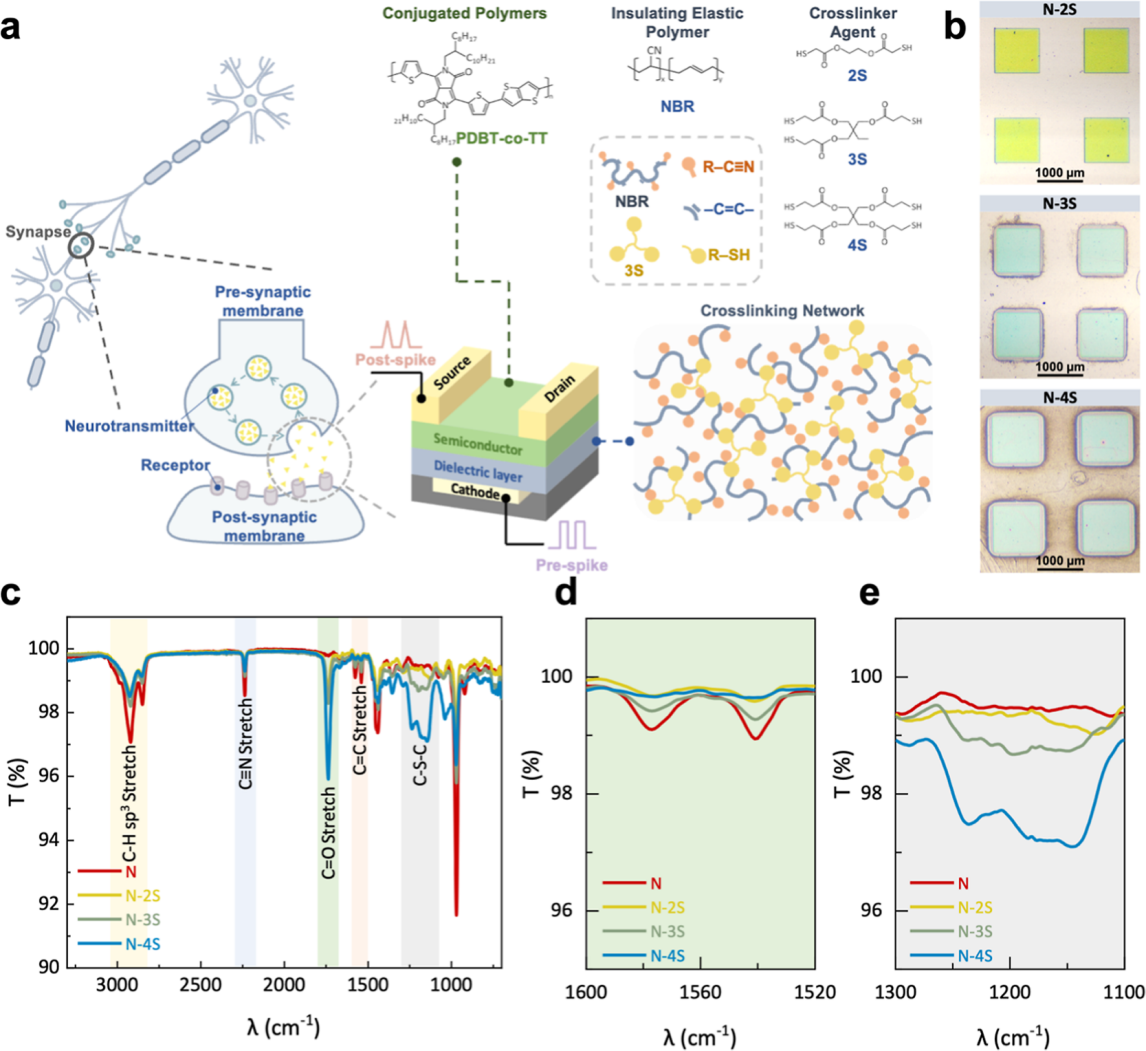
(a) Schematic diagram of synaptic transistors and chemical structures of the conjugated polymer, NBR, and thiol-based cross-linking agents. (b) Optical images of photopatterned NBR films with different cross-linkers, demonstrating photopatternability. (c) FTIR spectra show the characteristics peaks of pristine NBR and NBR with series cross-linkers after cross-linking. (d) Magnified view of the C=C stretching region, showing changes after cross-linking. (e) Magnified view of the C–S–C stretching region, confirming the formation of covalent bonds.

## 2 Experimental Details

### 2.1 Fabrications of Thiol–Ene Cross-Linked-Dielectric Layers

The elastic insulating polymer poly­(acrylonitrile-*co*-butadiene) (NBR, Aldrich, Mw = 198,952) was dissolved in *n*-butyl acetate (BAC) with a concentration of 50 mg mL^–1^ and heated overnight at 50 °C. The photoinitiator (TPO) and photo-cross-linking agent (2S, 3S, 4S) were also dissolved in BAC with a concentration of 40 mg mL^–1^. The solution was spin-coated at 1000 rpm onto an n-doped Si wafer. The coated film was then soft-baked on a 60 °C hot plate to remove most of the solvent. Subsequently, UV curing was performed using a UVACUBE 100 (Hönle, Germany) with UV light in the wavelength range of 315 to 400 nm (UVA) and a calibrated power of 5.4 mW cm^–2^ for 90 s to solidify the dielectric layer of the device. Gold electrodes with a thickness of 80 nm were deposited on the dielectric layer film using a thermal evaporation technique through a mask as metal–insulator–metal (MIM) capacitors.

### 2.2 AFM Analysis of Surface Morphology and Elastic Properties

Surface morphology and elastic modulus of the samples were analyzed using atomic force microscopy (AFM; Dimension Icon, Bruker, USA) operated in a PeakForce Tapping mode. Measurements were performed with a tip oscillation frequency of 2 kHz and a peak force limit of 10 nN. Force curves were recorded at each point within a 256 × 256 pixel grid. Elastic modulus was derived from each force curve using the DMT model.^200^ The AFM employed NPG-10 silicon nitride probes (Bruker) with a cantilever spring constant of 0.40 ± 0.20 N/m.

### 2.3 Fabrications of Field-Effect Transistors

The active layer was prepared using conjugated polymer PDBT-*co*-TT (Lumtec, Mw ∼ 30,000) dissolved in chlorobenzene with a concentration of 5 mg mL^–1^. The solution was spin-coated at 1000 rpm onto the dielectric layer, and then annealed at 150 °C for 1 h. Gold source and drain electrodes with a thickness of 80 nm were deposited on the semiconductor film using thermal evaporation. The channel length (*L*) and width (*W*) were 50 and 1000 μm, respectively. For the stretchable device, we first employed the blade-coating AgNWs as the stretchable gate electrode embedded in the spin-coated N-4S-based matrix. The active channel of the stretchable transistors is made from a stretchable polymer semiconductor blend (PDBT-*co*-TT/SBS) by using spin coating. This blend is composed of a high-mobility donor–acceptor conjugated polymer, PDBT-*co*-TT, and an elastic rubber, SBS, utilizing thiol–ene chemistry to achieve a semi-interpenetrating polymer network (SIPN). Sequentially, thermally deposited gold (Au) nanoparticles were employed on the elastic PDBT-*co*-TT/SBS layer to obtain drain-source electrodes. Through the combination of these intrinsically stretchable materials, we successfully fabricate the fully stretchable transistors for thiol–ene-cross-linked NBR-based dielectrics.

### 2.4 Electrical Characterization of Capacitors and Field-Effect Transistors

The capacitances of the MIM capacitors based on the dielectric layer with 2S, 3S, and 4S were measured using an LCR meter 4284A connected to a Keithley 2634B, and the dielectric constant (*k*) was calculated. The FETs were measured for their electrical properties using a Keithley 2634B semiconductor parameter analyzer inside a glovebox filled with argon gas. All dielectric properties were evaluated using 8 devices fabricated in the same batch, while all electrical characterizations of field-effect transistors were conducted on 6 devices from the same batch. The synaptic characteristics were measured by using the optimized devices.

### 2.5 Convolutional Neural Network Based on Mel Spectrogram Data Set

This acoustic data set consists of 21,000 samples generated from 10 simulated musical instruments, each producing 7 distinct pitches, recorded under 3 different acoustic conditions. For the acoustic classification task, a convolutional neural network (CNN) with a sequential architecture was used. The model begins with an input layer shaped according to the dimensions of the acoustic data, followed by 2 convolutional layers with 32 and 64 filters, respectively, both utilizing 3 × 3 kernels. Each convolutional layer is followed by 2 × 2 max-pooling layers, which progressively reduce spatial dimensions while extracting relevant features. Then, the feature maps are flattened and input to the fully connected layer (with 128 neurons) for classification. Finally, the output layer consists of 7 neurons corresponding to the 7 distinct pitches in the acoustic data set. The present CNN acoustic simulation applied synaptic weights are fitted based on the long-term potentiation (LTP) and long-term depression (LTD) in conductance change of actual synaptic transistors. CNN based on a Mel spectrogram acoustic data set was implemented and executed in a Python environment.

## 3 Results and Discussion

### 3.1 Morphology Characterizations of NBR-Based Dielectric OFET

The cross-linking reaction in the NBR dielectric layer is formed through a free-radical thiol–ene reaction. The free-radical thiol–ene addition involves the formation of radicals through light, heat, or free-radical initiators. These thiol radicals then attack the unsaturated double bonds in the NBR, forming a cross-linked structure. The reaction mechanism is illustrated in Figure 1a. Thiol–ene reactions are also known as thioetherification, where the –SH group in thiol–R-SH reacts with the C=C double bonds in the alkene (R_1_-C=C-R_2_), forming a thioether (R-S-R′). The thiol–ene reaction between NBR and thiol-containing cross-linkers (2S, 3S, and 4S) was successfully confirmed by Fourier transform infrared (FTIR) spectroscopy, as depicted in Figure 1c. The spectrum of unmodified NBR (N) exhibits characteristic peaks associated with its functional groups. Specifically, the peak at approximately 2240 cm^–1^ corresponds to the C≡N stretching vibration, attributed to the nitrile groups in NBR. The peaks in the 1540–1580 cm^–1^ region are attributed to the C=C stretching vibrations of the butadiene segments in NBR. Additionally, the peaks in the 2850–2950 cm^–1^ region represent the C–H stretching vibrations of sp^3^-hybridized carbon atoms in the polymer backbone. After the introduction of the thiol-containing cross-linkers, corresponding changes were observed in the FTIR spectra. The peak intensity associated with the C≡N stretching vibration decreased at 2240 cm^–1^, indicating that the nitrile groups may have participated in the cross-linking reaction. Additionally, compared to the uncross-linked pristine NBR film, the C=C peaks in the film were significantly weakened after adding the thiol cross-linkers (Figure 1d). This suggests that the C=C bonds have undergone changes due to reactions with the thiol groups in the cross-linkers. Furthermore, the new peak observed in the spectra of modified NBR samples (N-2S, N-3S, and N-4S) in the 1100–1250 cm^–1^ range can be attributed to the C–S–C stretching vibration, confirming the formation of covalent bonds between the thioether groups in the cross-linkers and the NBR polymer chains (Figure 1e). This observation provides direct evidence for the success of the thiol–ene reaction and the successful introduction of the cross-linkers into the NBR matrix.

Figure 1b shows the optical images of NBR films modified with different thiol-containing cross-linkers, highlighting the photopatterning achieved through the thiol–ene reaction and enhanced solvent resistance. In contrast, the uncross-linked film was removed by the solvent (Figure S1). The distinct square patterns observed on each film (N-2S, N-3S, and N-4S) indicate that such photopatterning technique provides precise control over the cross-linking process. This feature enables NBR films to be used for manufacturing large-scale device arrays, which is crucial for applications such as neuromorphic computing and sensors.

To investigate the effect of cross-linking density, AFM was employed to analyze the surface morphology of the thin films in tapping mode over a scanning area of 2 μm × 2 μm. Figure 2 shows the surface morphology of pristine and thiol–ene-cross-linked NBR layers. The uncross-linked pristine NBR layer (Figure 2a) has a relatively smooth surface with no well-defined morphology and the lowest surface roughness (0.265 nm). After adding thiol cross-linkers, the thiol–ene cross-linked N-2S layer (Figure 2b) exhibited distinct nanofiber-like features, indicating higher crystallinity and the highest surface roughness (0.559 nm) among all cross-linked films. As the thiol content increased, the nanofiber-like morphology of the cross-linked N-4S layer (Figure 2d) became less pronounced compared to the N-2S and N-3S layers (Figure 2c), but its surface roughness remains slightly higher (0.387 nm) than that of the uncross-linked pristine NBR layer. This morphological feature is attributed to the higher cross-linking density, as evidenced by the enhancement of C–S–C stretching peaks (Figure 1e), and the increased steric hindrance arising from the tetra-functional thiol cross-linker. These factors limit the polymer chain mobility and suppress the self-assembly of the N-4S film into obviously nanofiber-like features.

**Figure 2:**
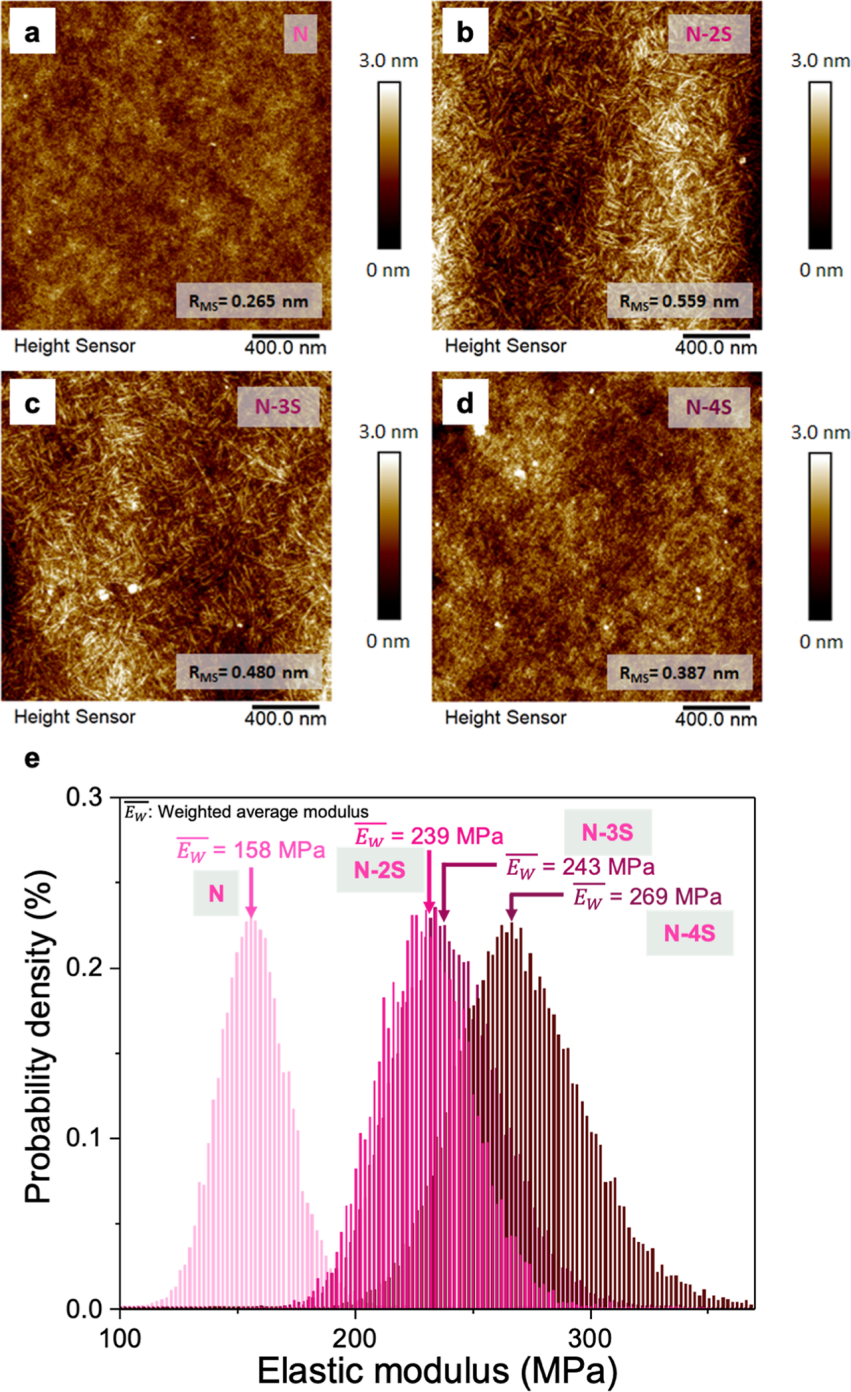
AFM morphology and elastic modulus distribution of NBR films using different thiol l cross-linkers. (a–d) AFM images of the surface morphology of NBR films using different cross-linkers (N, N-2S, N-3S, N-4S), highlighting differences in crystallinity and surface roughness. (e) Elastic modulus distribution of NBR films, indicating the influence of cross-linker type on film stiffness.

As depicted in Figure 2e, the distribution of elastic modulus for various thiol-cross-linked samples provides valuable insights into the mechanical properties of the dielectric layers. The uncross-linked NBR layer exhibited the lowest weighted average elastic modulus (*E*_w_ = 158 MPa), indicating it is the softest material. As the number of thiol functional groups increases (N-2S, N-3S, N-4S), the weighted average elastic modulus also increases (*E*_w_ = 239 MPa, 243 MPa, 269 MPa, respectively), indicating that the film becomes harder. This observation is consistent with the expectation that materials with higher cross-linking density are harder.

The observed differences in surface roughness significantly influence the electrical properties of the devices, with a higher surface roughness generally resulting in poorer electrical performance. The formation of nanofibers in the N-2S layer indicates that the polymer chains exhibit higher orientation due to their linear cross-linking structure. The higher roughness and distinct nanofiber morphology in N-2S may have a negative effect on its electrical applications. Conversely, although N-4S has the lowest surface roughness among the cross-linkers, it may facilitate charge transport of conjugated polymers on the N-4S surface.

### 3.2 Crystalline Characterizations of NBR-Based Dielectric OFET

Since a donor–acceptor diketopyrrolopyrrole (DPP)-based conjugated polymer (PDBT-*co*-TT) was utilized as the active layer in the device, its crystalline order in the layer deposited on different NBR dielectric layers (N, N-2S, N-3S, N-4S) was analyzed using grazing-incidence X-ray diffraction (GIXD). Figure 3 shows distinct high-order diffraction peaks, indicating the presence of ordered structures in the conjugated polymer films. The peak intensities varied with the dielectric layer, with N-4S exhibiting the smallest full width at half-maximum (fwhm), followed by N-3S, N-2S, and N. This suggests that the dielectric layer influences the crystal size of the conjugated polymer films. According to the Scherrer equation, we estimated the crystal sizes based on the fwhm. Figure 3c shows that the crystal size of the out-of-plane (200) peak increases from 141 to 189 Å with an increase in the cross-linking density of the dielectric layer. The increase in crystal size in the N-4S film may be attributed to the enhanced rigidity (Figure 2e), which promotes the crystallization of the conjugated polymers.

**Figure 3:**
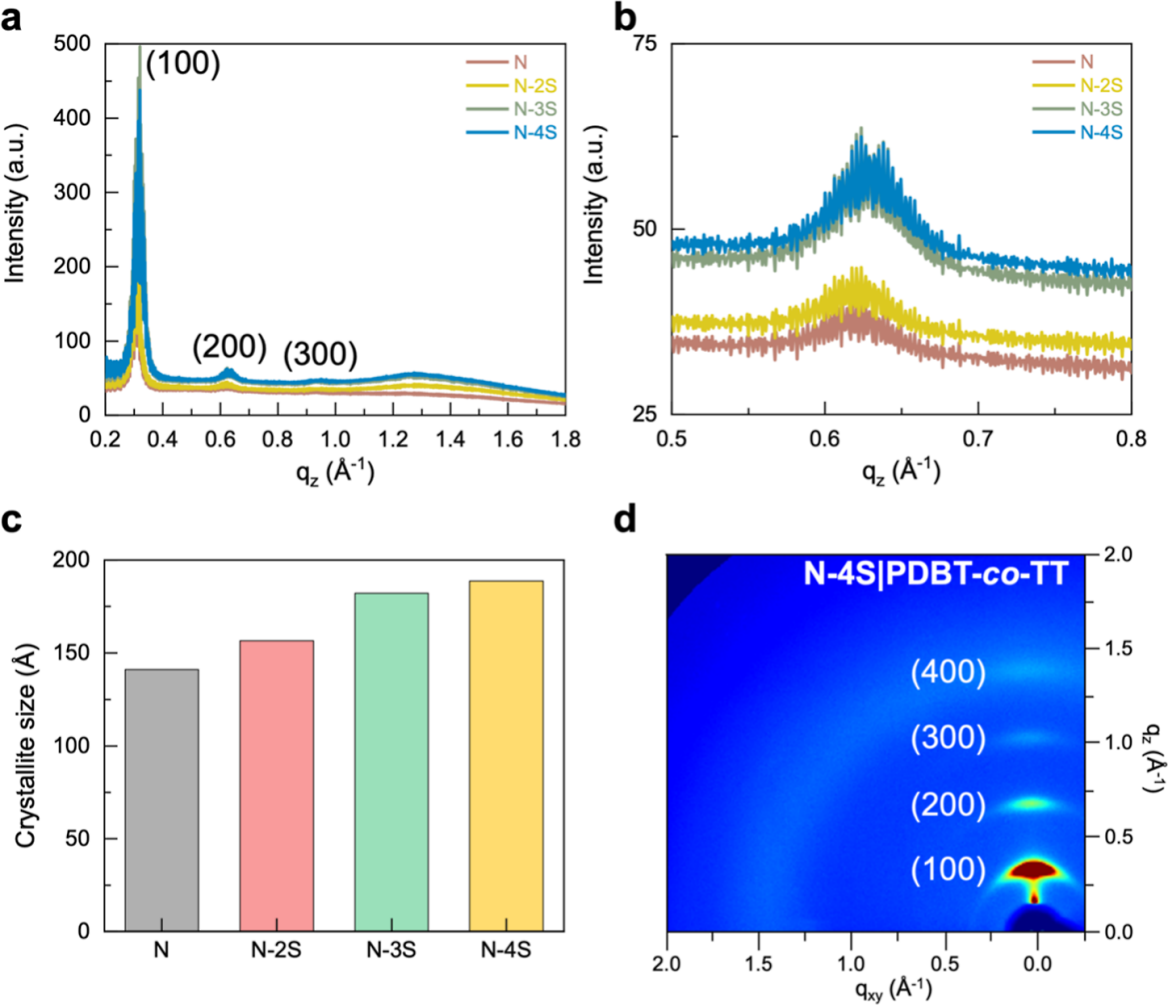
GIXD analysis of PDBT-*co*-TT films on different dielectric layers. (a) Out-of-plane 1D GIXD patterns showing diffraction peaks related to lamellar spacing and π–π stacking. (b) Intensity of the (200) peak, highlighting differences in peak width and intensity. (c) Crystallite size plots obtained from the (200) peak evaluation of PDBT-*co*-TT films on different dielectric layers. (d) 2D GIXD images of PDBT-*co*-TT on an N-4S dielectric layer, demonstrating enhanced diffraction intensity.

### 3.3 Electrical Performance of Stretchable NBR-Based Dielectric OFET

Initial characterization focused on the dielectric properties and capacitance of the NBR films with different thiol cross-linkers, measured using a MIM structure. Table 1 provides a comprehensive summary of the transistor performance and dielectric properties. The un-cross-linked pristine NBR dielectric layer exhibits the highest capacitance of 53.06 nF/cm^2^, while the photo-cross-linked N-2S, N-3S, and N-4S layers show relatively lower capacitances ranging from 25 to 31 nF/cm^2^. The lower capacitances of the cross-linked layers (N-2S, N-3S, N-4S) are primarily attributed to the increase in film thickness. Specifically, the thickness of the pristine NBR layer (N) was 215 nm, while the thickness of the thiol-modified films ranged from 389 (N-4S) to 448 nm (N-2S). This significant increase in the dielectric layer thickness is directly related to the decrease in capacitance. Notably, as the number of thiol functional groups increases, the dielectric constant of the cross-linked layers also increases, as shown in Figure 4a. Specifically, the thiol-cross-linked N-4S dielectric layer exhibits the highest dielectric constant (14.58), indicating that the introduction of thiol functional groups enhances charge storage capability.

**Table 1:** Electrical Properties of Field-Effect Transistor Using the Pristine NBR Layer and Different Thiol Crosslinking Agents as Dielectric Layers

dielectrics	*C*_*i*_ (nF/cm^2^)	thickness^Avg^ (nm)	*k* ^Avg^	μ_Lin._^Avg^ (cm^2^ V^–1^ s^–1^)	*I*_ON_/*I*_OFF_	*V*_TH_ (V)
N	53.06 ± 0.31	215	12.89	0.07 ± 0.01	10^2^	–0.7 ± 0.2
N-2S	25.85 ± 1.50	449	13.11	0.13 ± 0.03	10^3^	–1.4 ± 0.1
N-3S	31.03 ± 0.36	403	14.12	0.22 ± 0.02	10^3^	–0.7 ± 0.2
N-4S	31.35 ± 1.24	389	14.58	0.42 ± 0.04	10^4^	0.2 ± 0.4

**Figure 4:**
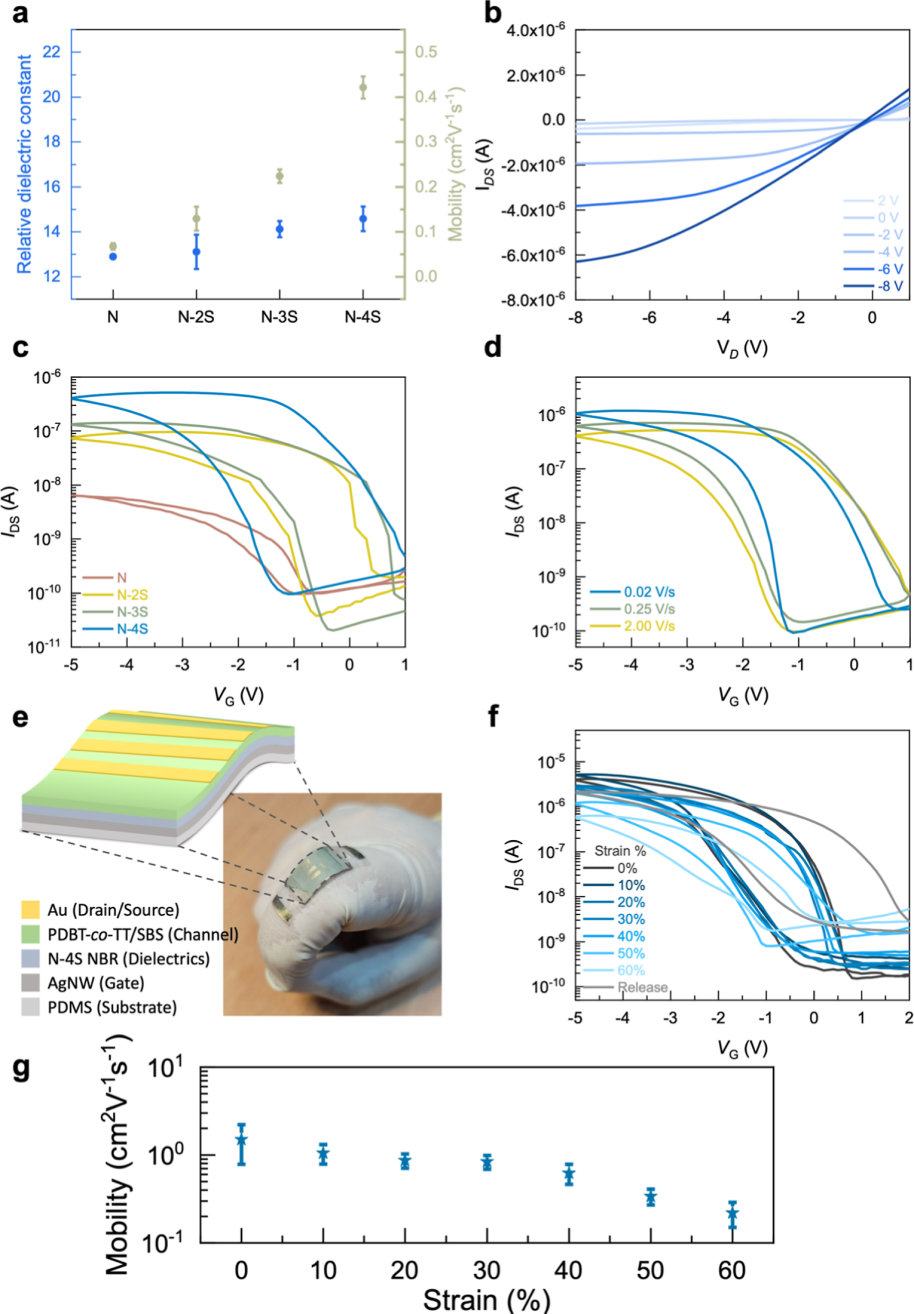
Electrical characterization of NBR-based dielectric devices. (a) Relative dielectric constants (*k*) of the different NBR dielectric layers and the charge carrier mobility of the corresponding devices. (b) Output characteristics of the N-4S device at various gate voltages. (c) Transfer characteristics of devices with different thiol cross-linking agents (N, N-2S, N-3S, N-4S) at a drain voltage (*V*_D_) of −5 V. (d) Transfer characteristics of the N-4S device at various gate voltage sweep rates. (e) Schematic diagram of a fully stretchable transistor device on a finger. (f) Transfer curves, (g) field-effect mobility of the stretchable transistor under different mechanical strains.

To investigate charge transport and memory properties, we prepare top-contact bottom-gate field-effect transistors using the pristine and thiol–ene-cross-linked NBR dielectrics. The donor–acceptor diketopyrrolopyrroles (DPP)-based conjugated polymer (PDBT-*co*-TT) was used as a semiconductor layer for charge transport. Figure 4b and S2 in the Supporting Information show the output characteristics of the NBR-based devices. The well-defined linear and saturation-region output characteristics suggest ohmic contact formation and effective channel modulation. Notably, all devices exhibit distinct p-channel transistor characteristics at a driving voltage of 5 V, indicating remarkable charge transport properties under a small driving voltage. Table 1 also summarizes the calculated results of the linear-regime charge carrier mobility (μ_lin_) for the fabricated devices. The mobility values were calculated using eq 1, the linear regime equation, and the obtained capacitance values. The charge carrier mobility (μ_lin_) can be calculated using the following formula, where *W*/*L* is the aspect ratio of the channel, *C*_*i*_ is the capacitance measured in the dielectric layer, and *V*_TH_ is the threshold voltage.

1 $$I_{DS}=\frac{W}{L}\mu_{lin}C_{i}\left(V_{G}-V_{TH}\right)V_{DS}$$

The mobility trend positively correlates with the number of thiol functional groups incorporated into the dielectric layer. Specifically, the N-4S device, possessing the highest number of thiol functional groups, exhibited the highest mobility (0.42 cm^2^ V^–1^ s^–1^). Conversely, the uncross-linked NBR device shows the lowest mobility (0.067 cm^2^ V^–1^ s^–1^). The trend in charge mobility is consistent with the GIXD results, which showed that the PDBT-*co*-TT film deposited on the N-4S dielectric layer has the largest crystal size. Furthermore, the increased mobility may also be attributed to the enhanced solvent resistance of the dielectric layer. High solvent resistance is advantageous for the fabrication of transistors. The pristine uncross-linked NBR device shows large leakage current and poor electrical stability, resulting in relatively lower device performance. Furthermore, the on/off current ratio of all devices ranges from 10^3^ to 10^4^, indicating good switching behavior. The threshold voltage (*V*_TH_) of all devices is in the range of −1.4 to 0.2 V, suggesting effective channel formation and low power consumption. Overall, the electrical characteristic analysis indicates that the photo-cross-linked N-4S dielectric layer exhibits the best transistor performance, with the highest charge carrier mobility while maintaining a good on/off ratio. More importantly, the clear correlation between the number of thiol functional groups, capacitance, and mobility suggests that the thiol–ene cross-linking chemistry plays a significant role in modulating the electrical performance of the devices.

The dual-swept transfer characteristics of devices fabricated using an uncross-linked NBR dielectric layer (N) and NBR-thiol–ene cross-linked dielectric layers (N-2S, N-3S, and N-4S) are presented in Figure 4c. All devices exhibited distinct hysteresis behavior. This current hysteresis is characterized by a shift in *V*_TH_ between the forward and reverse scans. In these devices, the dominant mechanism responsible for the observed hysteresis phenomenon is the orientational polarization of the NBR dielectric material. The hysteresis window is highly dependent on the content of thiol cross-linkers incorporated into the NBR matrix. Devices using the pristine NBR dielectric layer (N) showed the smallest hysteresis, indicating weak polarization effects. In contrast, devices using thiol–ene-cross-linked dielectric layers (N-2S, N-3S, and N-4S) display significantly larger hysteresis windows, suggesting enhanced polarization in the dielectric material. Notably, the N-4S device, which used the cross-linker with the highest number of thiol functional groups, exhibited the strongest hysteresis. This indicates that an increase in the number of thiol groups enhances the polarization effect within the NBR dielectric layer. The polar C–S bonds and nitrile groups promote this enhanced polarization, leading to a larger hysteresis window. This polarization-dominated hysteresis behavior holds significant potential for memory devices and artificial synapse applications. The nonvolatile nature of polarization enables gradual changes in channel conductivity, mimicking the dynamic behavior of biological synapses. The large hysteresis observed in the N-4S device is attributed to its strong dielectric polarization, making it a promising candidate material for applications requiring a wide operational window and tunable conductive states.

The dual-swept transfer characteristics of the device, particularly the N-4S device, exhibited noticeable hysteresis behavior, which is primarily attributed to the polarization of the NBR dielectric material, as mentioned earlier. Furthermore, after introducing thiol-based cross-linking (N-4S), noticeable changes in the *P*–*E* characteristics were observed (Figure S3), indicating an additional contribution to the polarization response. N-4S film exhibits a higher remanent polarization than the N film, resulting in stronger polarization retention, which is advantageous for realizing synaptic behavior under a lower electric field. Notably, the magnitude of the hysteresis window in the N-4S device is significantly correlated with the gate voltage scan rate, as illustrated in Figure 4d. Specifically, as the scan rate increases from 0.02 to 2.00 V/s, the hysteresis window of the N-4S device gradually widens. At slower scan rates, the dielectric polarization has more time to fully respond to changes in the gate voltage, resulting in a narrower hysteresis. Conversely, at faster scan rates, polarization lags behind the applied gate voltage, resulting in a wider hysteresis window. This enhanced hysteresis at higher scan rates further enhances the polarization dynamics in the N-4S dielectric layer. This scan rate-dependent hysteresis behavior is a characteristic of ferroelectric-like polarization in dielectric materials. The ability to tune the hysteresis window by adjusting the sweep rate provides additional control for potential memory and synaptic applications. For instance, different sweep rates can be used to program and erase memory states or to modulate synaptic weights in artificial synapses.

The enhanced hysteresis observed in the N-4S device, combined with its dependence on the gate voltage scan rate, further solidifies its potential for applications requiring tunable nonvolatile memory and bioinspired synaptic functionalities. The strong polarization characteristics of the N-4S dielectric layer are amplified at higher scan rates, offering a promising avenue for developing advanced electronic devices with customizable memory and learning properties. Further investigation into the underlying polarization mechanisms and optimization of the scanning rate for specific applications are crucial for realizing the full potential of these devices.

The N-4S dielectric layer exhibits optimal performance among NBR-based dielectric materials, featuring superior mechanical properties, solvent resistance, and a dielectric constant. This makes it highly suitable for both fully stretchable transistors and neuronal synaptic devices. To realize these all-stretchable transistors, the active channel is formed from a stretchable polymer semiconductor blend of PDBT-*co*-TT and an elastic rubber, styrene–butadiene–styrene block copolymer (SBS). This blend utilizes thiol–ene chemistry to achieve a SIPN. This SIPN semiconductor layer has been demonstrated to provide high charge transport properties and enhanced stretchability.^19^ Subsequently, thermally deposited gold (Au) nanoparticles were employed onto the elastic PDBT-*co*-TT/SBS layer to create stretchable drain-source electrodes. This approach is based on the principle that thermal deposition of gold nanoparticles on elastomeric substrates like SEBS can form a robust and highly stretchable conductive interface, known as a biphasic, nanodispersed (BIND) interface.^21^ Given that SEBS is a hydrogenated derivative of SBS, the application of thermally deposited gold electrodes on the SBS surface similarly forms a stretchable electrode. The gold/SBS electrodes exhibit low sheet resistance at low strains (0% to approximately 60%) (Figure S4). This indicates the great initial conductivity of the gold/SBS composite. However, a significant increase in sheet resistance is observed for the gold/SBS electrodes as the strain increases beyond 60%. Interestingly, we have found that the SBS film with cross-linking shows slightly lower sheet resistance at higher strains compared to the SBS film without cross-linking.

The schematic device structure of a fully stretchable transistor is shown in Figure 4e. Performance evaluation of this fully stretchable transistor under strain (Figure 4f,g) reveals that the all-stretchable N-4S device still maintains well-defined transfer curves even at 60% strain, with a charge carrier mobility of 0.22 cm^2^ V^–1^ s^–1^ and an on/off ratio exceeding 10^3^. The PDBT-*co*-TT/SBS films were characterized by GIXD (Figure S5), demonstrating robust crystallinity in the blend film under mechanical deformation. However, the charge carrier mobility decreases with increasing strain. The degradation of electrical performance is attributed to cracking of the thermal-deposited Au electrodes (Figure S6). Despite the decrease in charge mobility, it is noteworthy that the current hysteresis remains relatively stable when the strain is lower than 60%. This hysteresis stability suggests that the dielectric polarization mechanism responsible for the hysteresis behavior is not significantly affected. This characteristic is crucial for stretchable neuromorphic applications, as device performance remains consistent under mechanical strain.

### 3.4 Stretchable Neuromorphic Transistors Apply for Acoustic Recognition Network

The applicability of thiol–ene cross-linked NBR materials (N-2S, N-3S, and N-4S) for simulating neuronal synaptic functions was systematically investigated. Preliminary characterization revealed the presence of current hysteresis in N-2S, N-3S, and N-4S. The N-4S material has the largest hysteresis window, making it a prime candidate for artificial synaptic applications. We first used eq 2 to evaluate the paired-pulse facilitation (PPF) of synaptic devices using N-2S, N-3S, and N-4S dielectric layers.

2 $$PPF=\frac{A_{2}}{A_{1}}\times 100\%$$

where *A*_1_ and *A*_2_ represent the first and second excitatory postsynaptic currents (EPSC) calculated, respectively. As shown in Figure 5b, for all materials, the longer the pulse interval time, the lower the PPF percentage, which is consistent with the typical decay of short-term memory in biological synapses. Notably, N-4S exhibited superior short-term synaptic plasticity with a PPF as high as 242%, and its PPF response remained stronger than that of N-2S and N-3S across various pulse intervals. Moreover, under mechanical deformation, the N-4S-based stretchable synaptic transistor presented PPF values of 205.7% and 163.8% at strains of 0% and 60%, respectively. These considerable PPF values are attributed to the strong orientational polarization of the N-4S dielectric layer, which enhances carrier modulation at the semiconductor/dielectric interface. Figure 5c presents the characteristics of spike-width-dependent plasticity (SWDP) to independent spikes of different durations. All N-series devices showed a sustained increase in current with an increasing duration time. These results indicate that synapses can be modulated by controlling the duration of time. Consistent with the overall device performance, the N-4S device produced the highest current under the same stimulus duration. This suggests that the N-4S material offers a broader dynamic range for representing synaptic weights. Figure 5d investigates the device response to repetitive stimulation at different frequencies, particularly for the N-4S device. The figure shows the current amplitude after 10 consecutive pulses. Figure 5e further simulates synaptic learning characteristics based on the N-4S device with multiple spikes. The results show that increasing the number of stimulus pulses (from 5 to 100) leads to an increase in the EPSC and a prolongation of the decay time after stimulus removal. This behavior aligns with the concept of synaptic consolidation, where repeated stimulation reinforces memory traces, demonstrating that the device possesses learning and memory retention capabilities that are dependent on the stimulation history.

**Figure 5:**
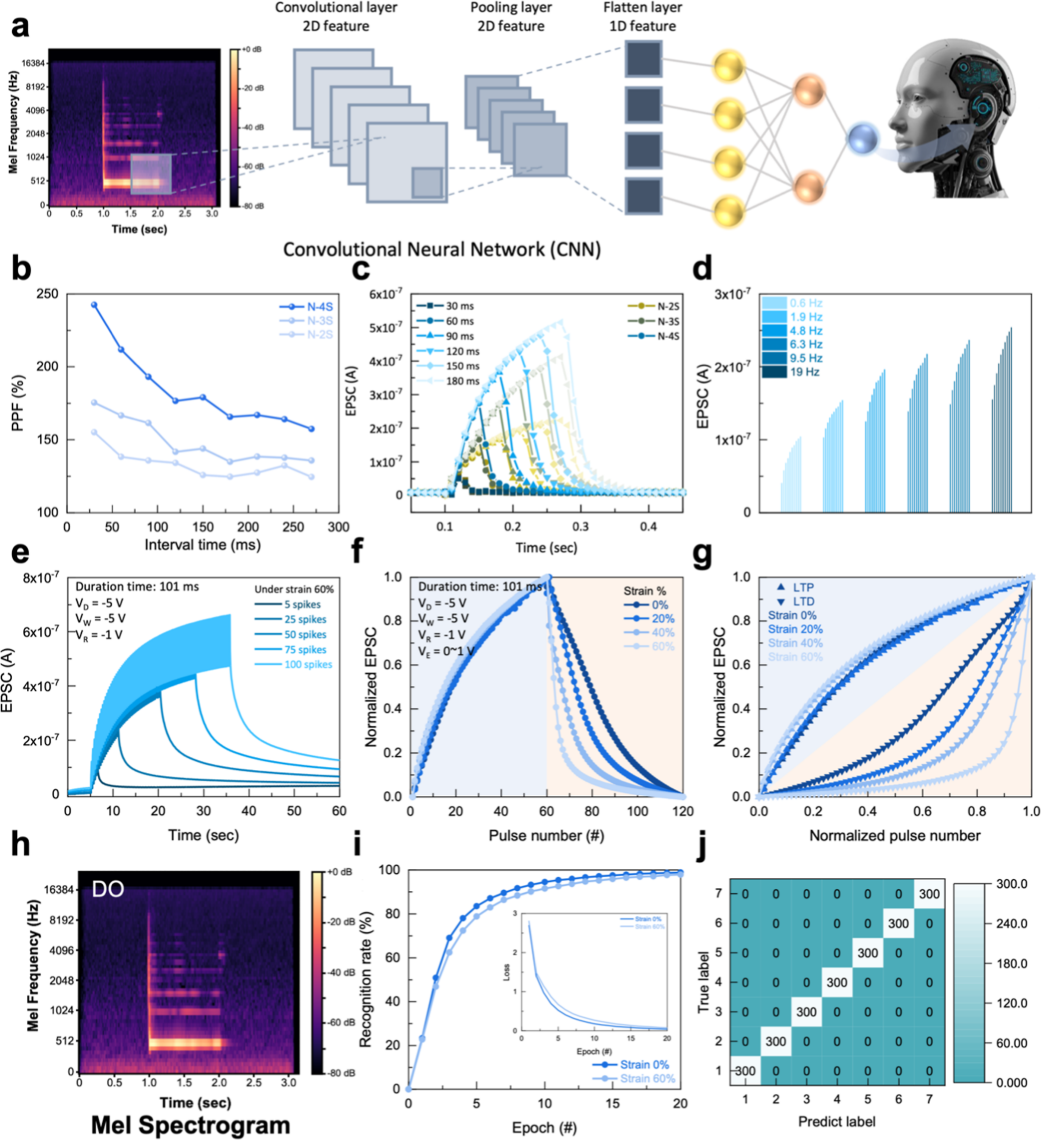
(a) Schematic diagram of stretchable NBR-based synaptic transistors in neuromorphic computing. Synaptic characteristics of NBR-based dielectric devices: (b) PPF percentage as a function of pulse interval time for devices with N-2S, N-3S, and N-4S dielectric layers. (c) SWDP responses to independent spikes of varying durations (30 to 180 ms) for N-2S, N-3S, and N-4S devices. (d) The current response of the N-4S device after 10 consecutive stimuli at different frequencies. (e) Spike-number-dependent plasticity showing the response of the N-4S device to increasing numbers of consecutive stimuli under 3.24 Hz. (f) Demonstration of LTP and LTD in the unstretched and stretched N-4S device. (g) Normalized conductance curves corresponding to the LTP/LTD behavior shown in (f), plotted against normalized pulse number, illustrating nonlinear characteristics of synaptic weight. (h) Mel-frequency mapping based on synthesized pitches generated by virtual instruments. Distinct horizontal bands suggest harmonic overtones and tonal characteristics. Training results of CNN model under mechanical strain conditions (0% strain and 60% strain). (i) Recognition accuracy, and insert the training loss plots based on the N-4S device under mechanical strain conditions. (j) Confusion matrix for the classification task of 7 categories (300 samples per class) at 60% strain. In synaptic behavior characterization, the electrical modulated parameters are drain voltage (*V*_D_ = −5 V), writing voltage (*V*_W_ = −5 V), reading voltage (*V*_R_ = −1 V), and erasing voltage (*V*_E_ = 0–1 V), respectively.

Figure 5f,g illustrate the significant impact of mechanical strain on the device’s LTP and LTD behaviors, which are crucial for simulating synaptic plasticity in neuromorphic applications. It clearly demonstrates that repeated writing pulses lead to a gradual increase in current (synaptic strengthening), while subsequent erasing pulses result in a progressive decrease in current (synaptic inhibition). This behavior confirms that the device can modulate its conductive state in response to stimuli, mimicking the weight changes of biological synapses, which is essential for neuromorphic computing. Figure 5g presents the fitting curves derived from the data in Figure 5f, with a normalized pulse number as the horizontal axis. This normalization allows for the assessment of the nonlinearity (NL) of synaptic weight updates during LTP and Ltd. Ideally, to achieve predictable analog computation, these curves should approach linearity. The curves representing the unstretched device based on this figure show a more reasonable linear transition. In contrast, compared to the unstretched device, the device subjected to 60% strain exhibited higher NL in the LTP/LTD responses. This result is quantitatively supported by fitting parameters obtained using the Ebbinghaus forgetting curve adaptation method. At 60% strain, the absolute values for the NL metrics for LTP and LTD are both greater than those at 0% strain (NL values change from 2.31 to 2.72 for LTP and from −2.1 to −7.42 for LTD, respectively, when comparing 0% and 60% strain). The LTD of strain 60% showed rapid conductance decay due to cracks of formation in the Au electrodes (Figure S6). Therefore, improving the stretchability of the electrode is crucial to achieving reliable stretchable devices. Several stretchable electrode materials, including spray-coated carbon nanotube,^22^ stretchable PEDOT films, have been reported as potential alternatives.^23,24^ Despite the increase in NL, the characteristics of LTP and LTD were preserved even under a significant 60% strain. This indicates that the N-4S-based stretchable synaptic transistor exhibits a mechanical robustness and functional stability. This resilience is crucial for achieving reliable wearable neuromorphic applications.

To demonstrate the potential of neuromorphic applications, we combined a proprietary acoustic data set and a CNN model with the tunable synaptic weights of the N-4S device for a proof-of-concept demonstration. We simulated the device for its proficiency in differentiating discrete pitch stimuli (DO, RE, MI, FA, SO, LA, and TI). This fundamental perceptual task, critical for auditory scene analysis and language comprehension, serves as a key benchmark for emulating essential neural processing mechanisms. Audio signals were captured by using a commercial recorder in a controlled environment. The raw waveforms were preprocessed and converted into Mel-frequency cepstral coefficients to construct a data set for training and evaluating neuromorphic devices, as shown in Figures 5h and S8.

We applied the synaptic weights obtained under unstretched (0% strain) and mechanical deformation (60% strain) conditions to the acoustic CNN model. As shown in Figure 5i, even after mechanical deformation, the synaptic transistors maintained an excellent classification performance, with a recognition accuracy rate approaching 99%. Subsequently, we used the handwritten digits in the MNIST data to validate the same CNN model based on N-4S device parameters. This MNIST-based simulation achieved classification accuracies of 93.71% and 92.51% (Figure S9). These results confirm the validity of our device’s synaptic characteristics and CNN model. The powerful 2D feature learning capability of the CNN architecture and the acoustic features with distinguishable pitch detail are attributed to realizing the high accuracy. To further evaluate the classification reliability of the CNN model based on stretchable synapses, we performed a confusion matrix analysis to assess the model performance on unseen test data.

As shown in Figure 5j, the results reveal a distinct diagonal alignment in the chart, indicating that the model maintained a high pitch recognition accuracy even at 60% strain. This highlights the stability and accuracy of the proposed synaptic device and CNN architecture. In summary, our synaptic device demonstrates stable and tunable weight behavior under mechanical strain, paving the way for fully stretchable neuromorphic applications.

## 4 Conclusions

This work successfully developed and characterized a stretchable, high-*k* dielectric material based on NBR cross-linked via thiol–ene chemistry for neuromorphic applications. By engineering the NBR with various thiol functional groups, such as N-4S, we achieved a material platform combining a high dielectric constant with great elasticity and a photopatterning capability. Furthermore, unlike traditional photolithography, our NBR material acts as its own photoresist. The UV-exposed areas become cross-linked and insoluble, while the unexposed areas can be removed easily with a solvent. This eliminates several complex steps (e.g., separate photoresist coating, etching, and stripping), which simplifies the manufacturing flow, reduces materials waste, and lowers production costs. This enables the fabrication of a transistor to operate at low voltage and is compatible with conventional photolithography and scalable for large-area neuromorphic array fabrication. Furthermore, a large current hysteresis driven by dielectric polarization was observed, particularly in N-4S, indicating its potential for neuromorphic computing. The N-4S device exhibited critical biological synaptic functions, including short-term and long-term plasticity, and demonstrated memory and learning capability. The device fabricated with the fully stretchable transistor using stretchable NBR dielectrics can maintain excellent electrical performance and stable hysteresis, even under high strain (up to 60%). Notably, the device can still perform basic synaptic behaviors such as LTP and LTD under 60% strain. For practical neuromorphic applications, the tunable synaptic weights of the N-4S device (both unstretched and at 60% strain) have been successfully integrated with an acoustic CNN model. This stretchable synaptic transistor demonstrated excellent classification performance, achieving recognition accuracy close to 99% even under severe mechanical deformation. This sustained high accuracy highlights the robust feature learning capability of combining the CNN architecture with the stable and tunable weight behavior of the stretchable synaptic device. This work presents a critical step toward realizing fully stretchable neuromorphic circuits essential for advanced wearable electronics and artificial intelligence.

## Supplementary Material



## Notes

Optical images of un-cross-linked film, Au/SBS film, and cracked electrodes; output characteristics of a series of devices; polarization-electric field curve of the dielectric film; GIXD analysis of stretchable polymer semiconductor; PPF of cyclic stretch; feature images (RE, MI, FA, SO, LA, and TI) of acoustic data set; and training results of CNN model based on MNIST data set (PDF)
