# Supplementary material for: Polarizable Thiol–Ene Cross-Linked Nitrile Dielectrics for Stretchable Low-Voltage Neuromorphic Transistors with Acoustic Classification
Source: ACS Appl Mater Interfaces. 2026 Jan 5;18(1):2188–99. doi: 10.1021/acsami.5c18342 (PMC12781051; doi:10.1021/acsami.5c18342)
Supplement: Supplementary file 1 [file am5c18342_si_001.pdf]

(Supporting Information)

Polarizable Thiol-ene Crosslinked Nitrile Dielectrics for Stretchable Low-Voltage Neuromorphic Transistors with Acoustic Classification

Chang-Jing Liu,[1,‡] Shu-Wei Hsiao, [1,‡] Qun-Gao Chen, [1,‡] Qi-An Hong,[3] Yen-Ting Lin,[4] Chu-Chen Chueh,[5] Chan-Tat Ng,[6] Ting-Ting Chang,[6] Seong H. Kim,[4,] Yu-Cheng Chiu,[3,*] and Wen-Ya Lee[1,2,*]*

[1] Department of Chemical Engineering and Biotechnology, National Taipei University of Technology, Taipei 10608, Taiwan

[2] High-Value Biomaterials Research and Commercialization Center, National Taipei University of Technology, Taipei 10608, Taiwan

[3] Department of Chemical Engineering, National Taiwan University of Science and Technology, Taipei 10607, Taiwan

[4] Department of Chemical Engineering and Materials Research Institute, The Pennsylvania State University, Pennsylvania 16802, United States

[5] Department of Chemical Engineering, National Taiwan University, Taipei 10617, Taiwan

[6] Department of Psychology and Research Center for Mind, Brain & Learning, National Chengchi University, Taipei 11605, Taiwan

‡ These authors contributed equally.

Corresponding author email: wenyalee@mail.ntut.edu.tw; ycchiu@mail.ntust.edu.tw; shk10@psu.edu

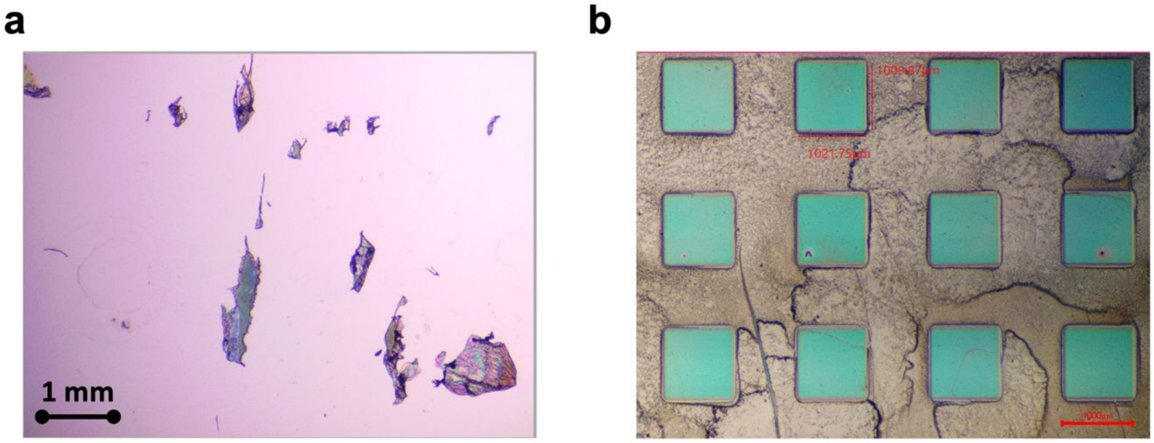

Figure S1. Optical images of N-4S film: (a) uncrosslinked film and (b) photo-patterned film, were immersed in chlorobenzene for 30 sec, respectively.

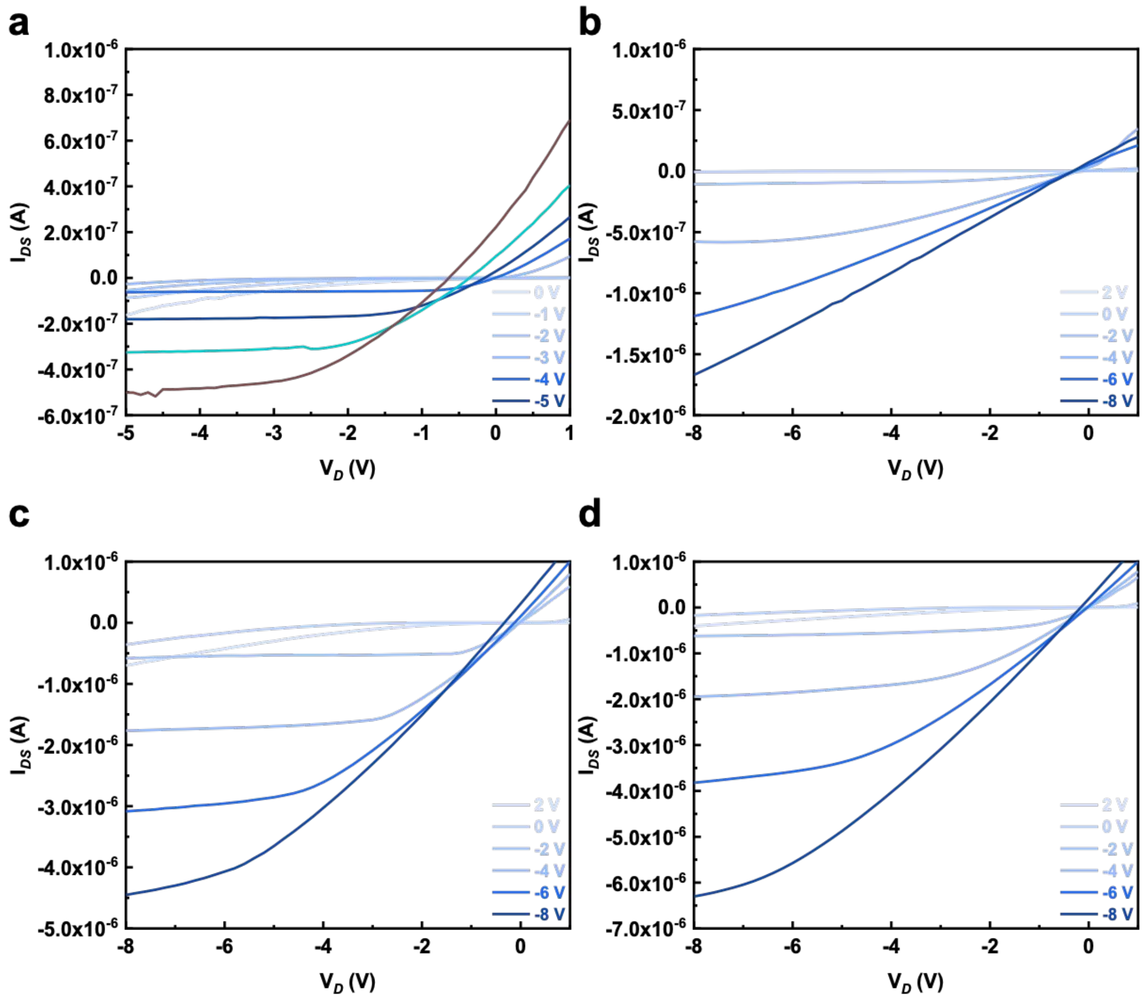

Figure S2. Output characteristics of the pristine and thiol-ene-crosslinked NBR-based dielectric devices with different thiol crosslinkers. (a) Pristine NBR, (b) N-2S, (c) N-3S, and (d) N-4S.

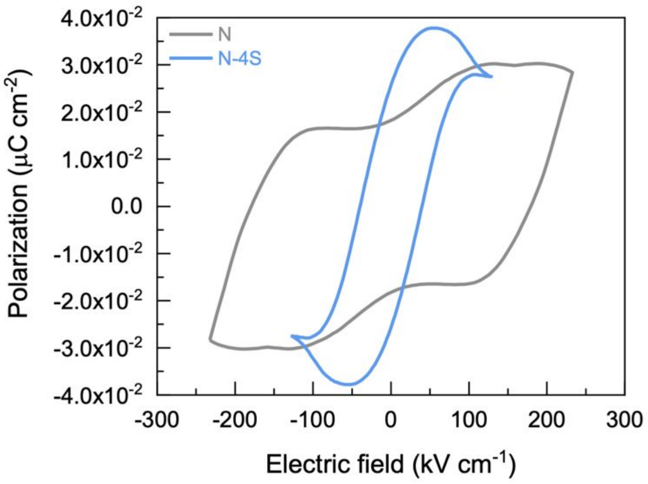

Figure S3. The polarization-electric field hysteresis loop of N film and N-4S film.

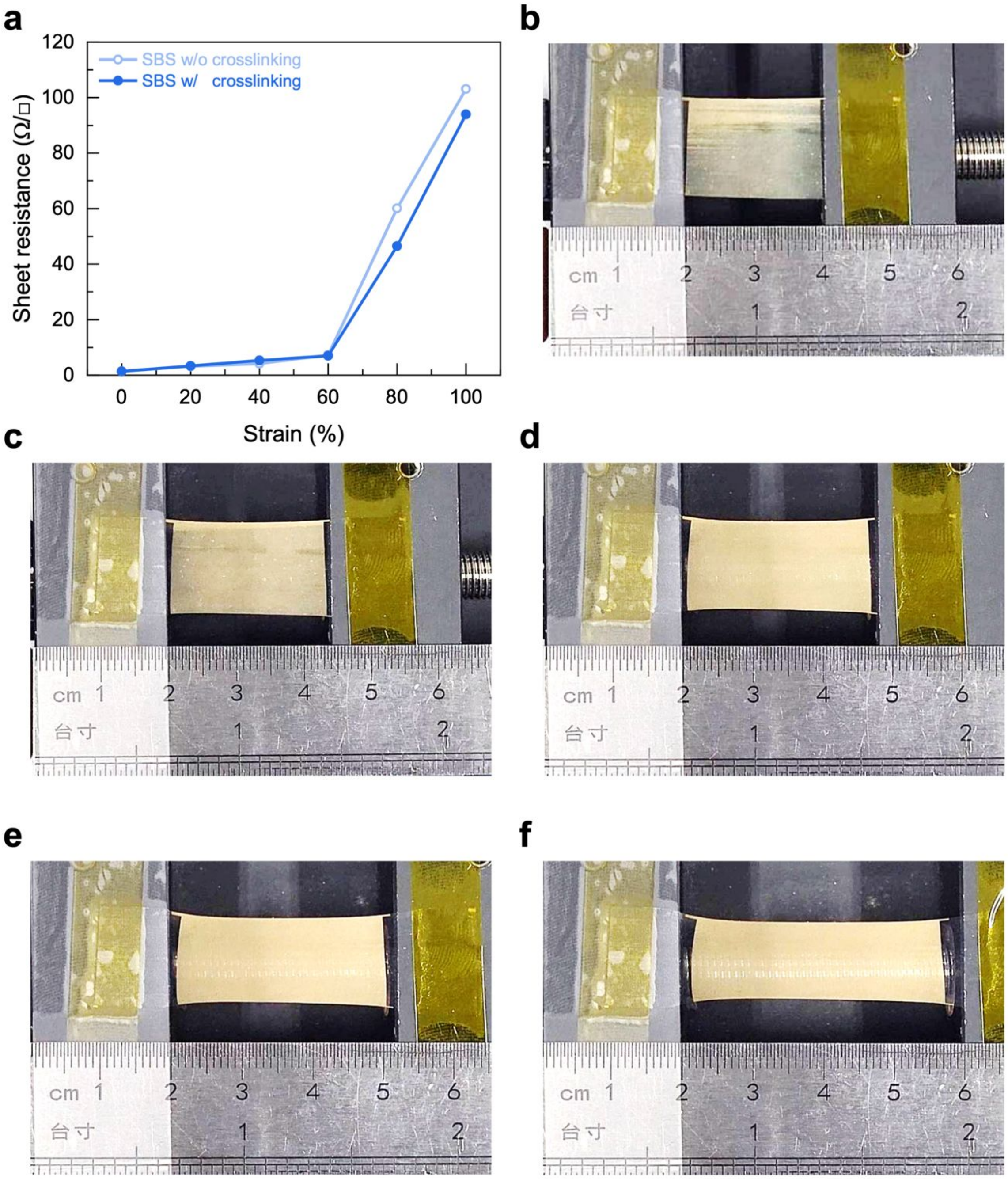

Figure S4. (a) Sheet resistance as a function of strain for SBS films with and without crosslinking. Optical images of stretchable Au/SBS electrodes under various strains: (b) 0%, (c) 20%, (d) 40%, (e) 60%, and (f) 100%.

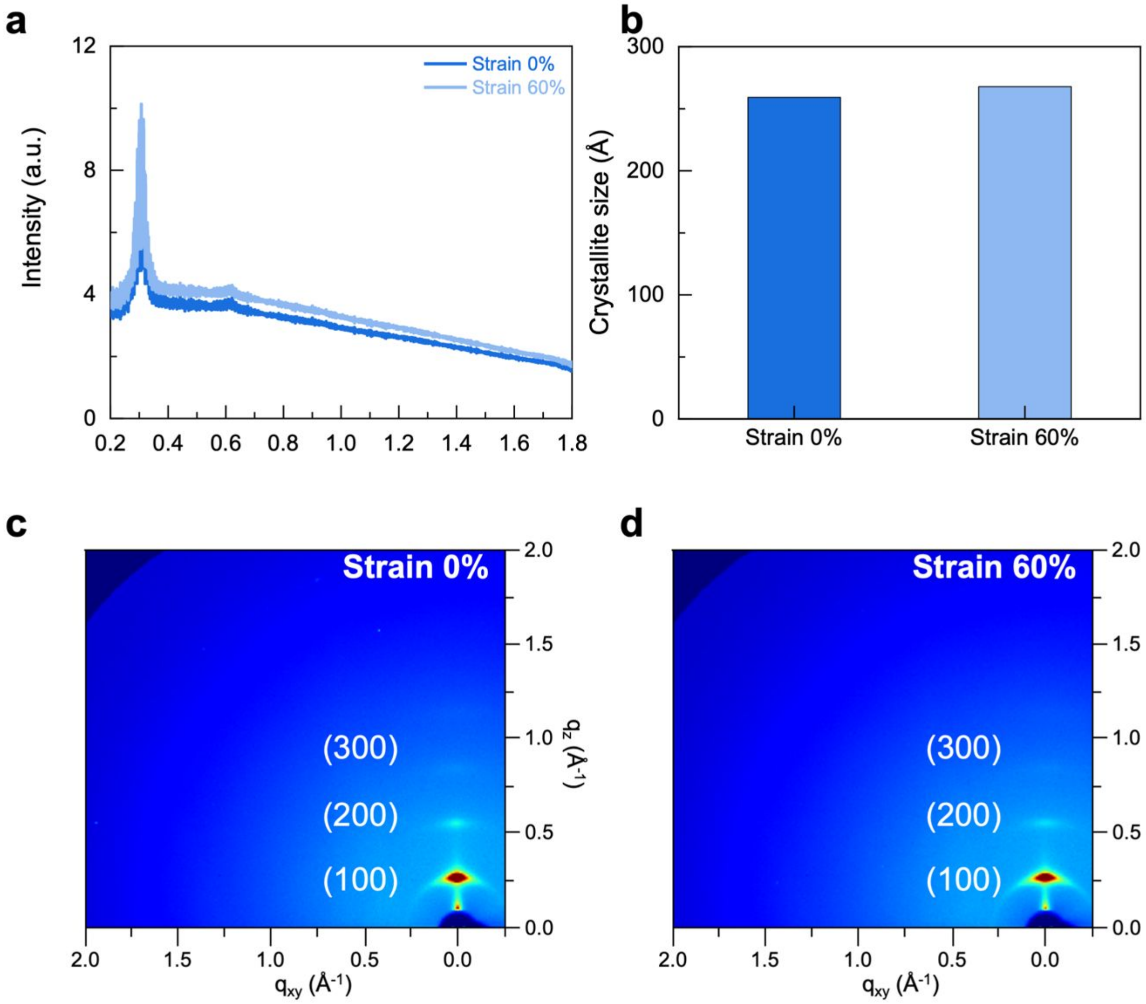

Figure S5. GIXD analysis of PDBT-*co*-TT/SBS composite films. (a) Out-of-plane 1D GIXD curves. (b) Crystallite size plots obtained from the (100) peak evaluation of strain 0 and 60%. 2D GIXD images of PDBT-*co*-TT/SBS composite films under (c) strain 0% and (d) strain 60%.

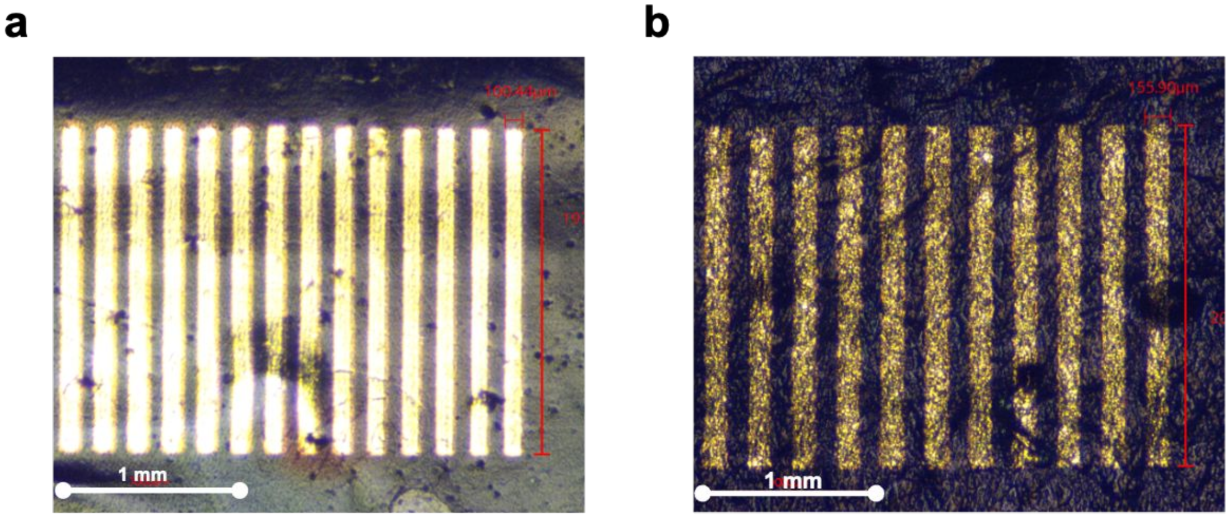

Figure S6. Optical images of Au electrodes on the all-stretchable transistors: (a) 0% strain and (b) 60% strain.

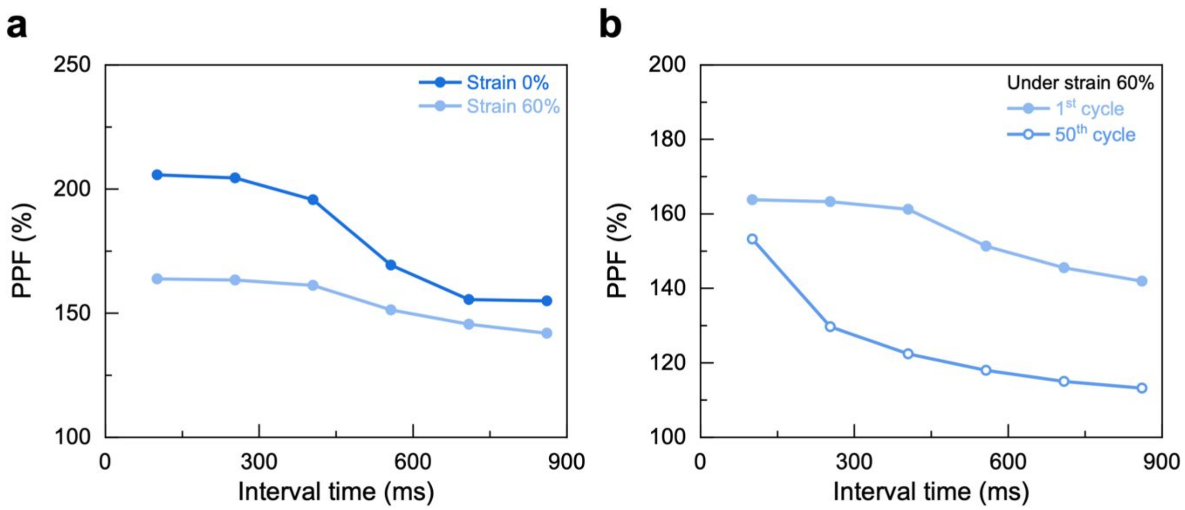

Figure S7. Paired-pulse facilitation (PPF) percentage as a function of pulse interval time. The stretchable synaptic transistor measured (a) mechanical deformation, and (b) 50 cycles of mechanical deformation under strain 60%.

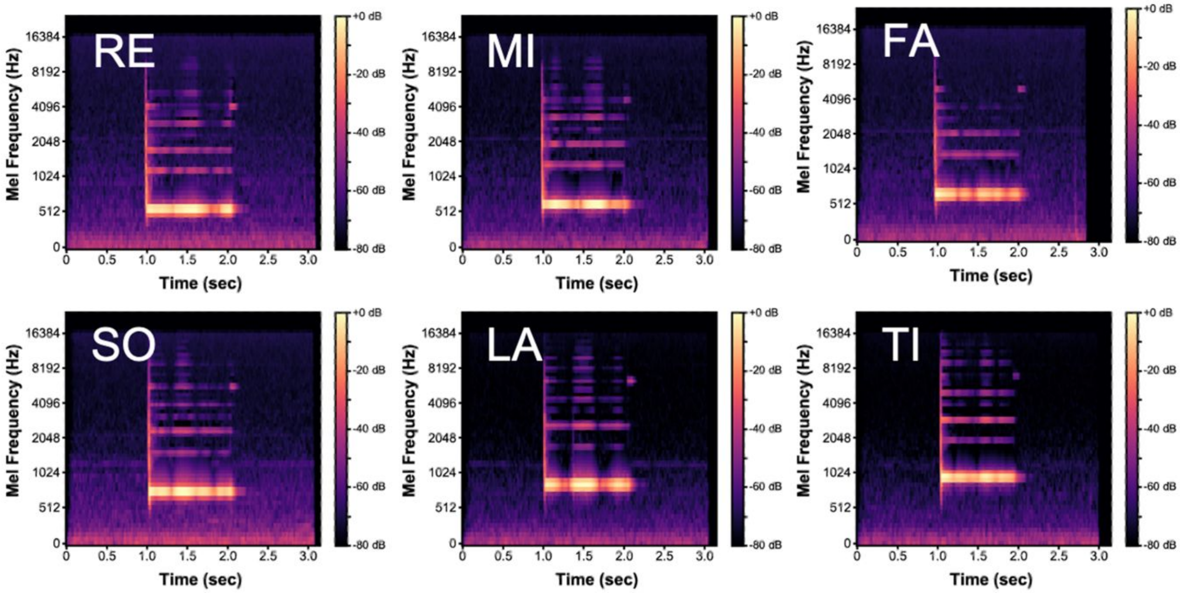

Figure S8. Representative feature images utilized virtual instruments to generated different synthesized pitches (RE, MI, FA, SO, LA, TI).

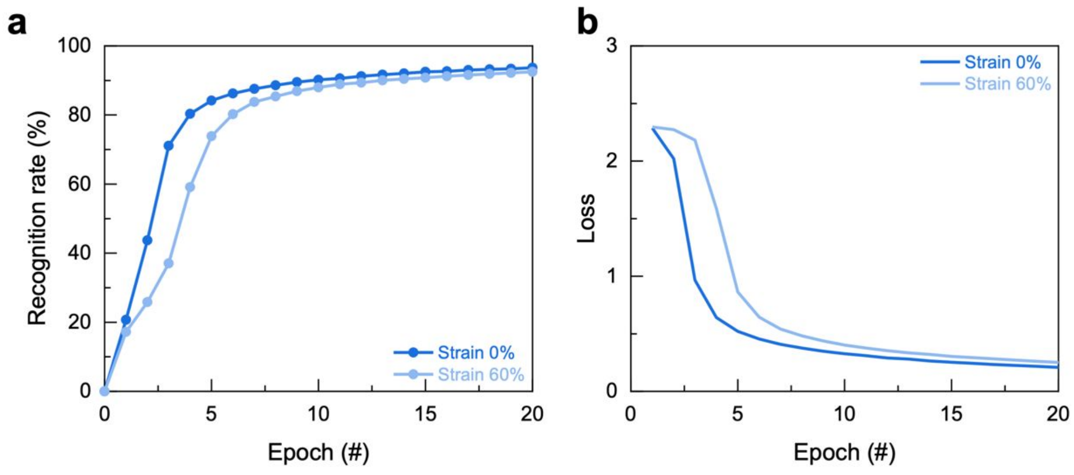

Figure S9. Training results of CNN model under mechanical strain conditions (0% strain and 60% strain). (a) Recognition accuracy, and (b) training loss plots based on the N-4S device under mechanical strain conditions.
